# The Construction and Characterization of an Infectious Clone of the Asian Genotype Chikungunya Virus from Yunnan, China

**DOI:** 10.3390/tropicalmed10100278

**Published:** 2025-09-26

**Authors:** Xinhang Ning, Binghui Xia, Zimeng Cheng, Liuyi Zhang, Fengfeng Mo, Hao Ren, Hailin Tang

**Affiliations:** 1 Department of Biomedical Protection, Faculty of Naval Medicine, Naval Medical University, Shanghai 200433, China; m15048080669@163.com (X.N.); xbhnjucpu@163.com (B.X.); 13582818881@163.com (Z.C.); greatlisa@163.com (L.Z.); 2 Key Laboratory of Biological Defense, Ministry of Education, Second Military Medical University, Shanghai 200433, China; 3 Shanghai Key Laboratory of Medical Bioprotection, Second Military Medical University, Shanghai 200433, China; 4 Department of Naval Nutrition and Food Hygiene, Faculty of Naval Medicine, Naval Medical University, 800 Xiangyin Road, Shanghai 200433, China; mofengfeng@smmu.edu.cn; 5 Department of Microbiology, Faculty of Naval Medicine, Navy Medical University, 800 Xiangyin Rd, Shanghai 200433, China

**Keywords:** Chikungunya virus, Asian genotype, infectious clone, reverse genetics

## Abstract

Chikungunya fever (CHIKF), which is caused by the Chikungunya virus (CHIKV), has rapidly spread across the globe in recent years, leading to its listing as a public health concern by the World Health Organization. In 2019, the first local outbreak of Asian-type CHIKF was reported in Xishuangbanna, Yunnan Province, China, with 88 CHIKV nucleic acid-positive cases detected from clinical specimens. To further investigate the biological characteristics of the virus strain responsible for this outbreak, we reconstructed the 625D6h strain using reverse genetics and tested its growth kinetics in different cell lines. The results showed a strong replication capacity in the *Aedes albopictus* C6/36 insect cell line but a weaker one in mammalian cell lines. The virus’s high replication capacity in mosquito cells is an interesting phenotype that warrants further study to determine if it influences vector adaptation and transmission dynamics in endemic settings. The study also found that two DHODH inhibitors, ML390 and vidofludimus, could effectively inhibit CHIKV replication in vitro. The infectious clone created in this study provides a useful tool for studying the recently prevalent Asian-type strain in China, supporting the subsequent development of prevention and treatment methods.

## 1. Introduction

Chikungunya virus (CHIKV) is a single-stranded positive-sense RNA virus belonging to the genus *Alphavirus* within the *Togaviridae* family. It is transmitted to humans through *Aedes albopictus* or *Aedes aegypti* mosquito bites, which cause Chikungunya fever (CHIKF) [1]. CHIKV was first isolated from a febrile patient in Tanzania in 1953 [2]. Prior to 2004, it was only prevalent in Africa and Southeast Asia. In 2004, eastern Kenya saw a CHIKF outbreak that subsequently expanded into the surrounding areas of the Indian Ocean. Currently, the disease is prevalent in multiple regions, including Asia, Africa, Europe, and the United States, affecting millions of people [3,4]. As of July, since the beginning of 2025, 16 countries/regions have reported approximately 240,000 cases of CHIKF and 90 CHIKV-related deaths. Currently, the Americas is the region with the highest number of reported CHIKV infections globally. As of 15 July 2025, Brazil reported 185,553 cases, making it the country with the highest number of reported CHIKV infections this year. According to the World Health Organization’s risk assessment, CHIKV has become a potential risk affecting public health [5].

The CHIKV genome is approximately 11.8 kb in length, with a virion diameter of about 70 nm. It contains two open reading frames (ORFs) encoding four nonstructural proteins (nsP1, nsP2, nsP3, and nsP4) and five structural proteins (C, E3, E2, 6K, and E1) [6,7,8]. Following entry into target cells via endocytosis, CHIKV synthesizes nsP polyproteins from its positive-sense RNA. These polyproteins are subsequently cleaved to form the nsP1-4 replication complex, which facilitates the synthesis of negative-strand RNA [9]. nsP1 possesses methyltransferase activity involved in mRNA capping [10]. nsP2 and nsP3 function as RNA helicase and replicase, respectively, in viral replication, while nsP4 serves as the RNA-dependent RNA polymerase [11]. The negative-strand RNA acts as a template for both the genomic RNA and the 26S subgenomic RNA, with the latter responsible for structural protein expression. Viral RNA assembles with the nucleocapsid protein (C) to form the viral core, which then buds from the plasma membrane after envelope protein (E) incorporation to produce virions [12]. The envelope glycoproteins E1 and E2 initially form heterodimers, which subsequently organize into trimeric spikes embedded in the viral envelope, mediating receptor binding, attachment, and membrane fusion [13]. The E3 protein facilitates pE2 (E2 precursor) folding and stabilizes the E2-E1 spike complex [14].

CHIKV is classified into four genotypes: West African (WA), East/Central/South African (ECSA), Asian, and Indian Ocean lineage (IOL) [15]. The virus is believed to have originated in Central/East Africa approximately 300–500 years ago [16,17]. Initially confined to eastern, central, and southern Africa, CHIKV subsequently diverged and spread to other regions [17,18]. Divergence between ECSA and Asian lineages occurred around 150 years ago (between 1879 and 1927), leading to multiple outbreaks in Southeast Asia and India [19]. Notably, the Asian lineage was responsible for the first CHIKV outbreak in the Caribbean in late 2013 [20], which rapidly spread across Pacific islands and much of the Americas, causing numerous epidemics [21,22]. During the 2005–2006 Réunion Island outbreak, the ECSA lineage evolved into the IOL subgenotype [23].

In 2019, China’s Yunnan Province reported its first Asian-genotype CHIKV outbreak, with 88 nucleic acid-positive cases detected, including four with full-genome sequencing [24]. Reverse genetics technology, which enables the direct manipulation of viral genomes in vitro to rapidly synthesize wild-type or mutant strains, has significantly advanced research on viral infection, transmission, and pathogenesis, serving as a crucial tool in virology [25]. This technology also facilitates the development of live-attenuated vaccines, contributing to serodiagnosis and antiviral screening [26]. To further investigate the infectivity and pathogenic characteristics of the Asian-genotype CHIKV strain circulating in Yunnan in 2019, we successfully constructed an infectious clone of the 625D6h strain (GenBank: OK316995.1). Using this infectious clone, we analyzed its infection properties in mammalian and mosquito cells, evaluating the antiviral effects of two compounds.

## 2. Materials and Methods

### 2.1. Cell Culture

The baby hamster kidney cells (BHK-21, ATCC CCL-10), human rhabdomyosarcoma cells (A-673) (ATCC CRL-1598), U2-OS cells (ATCC HTB-96) and African green monkey kidney (Vero, ATCC CCL 81) were obtained from ATCC (American Type Culture Collection, Manassas, VA, USA). The immortalized human skeletal muscle cells were purchased from Shanghai Fuheng Biotechnology Co., Ltd. (Shanghai, China), with the catalog number FH-Y033. The C6/36 *Aedes albopictus* cells (ATCC CRL-1660) were purchased from Wuhan Punosai Life Science Technology Co., Ltd. (Wuhan, Hubei, China), with the catalog number CL-0504. The *Aedes aegypti* cell line Aag-2 was kindly provided by Professor Sibao Wang, CAS Center for Excellence in Molecular Plant Sciences. All mammalian cells—including BHK-21, Vero, U2-OS, A-673, and human skeletal muscle cells—were cultured at 37 °C with 5% CO_2_. The culture medium consisted of DMEM (Gibco, Waltham, MA, USA) supplemented with 10% fetal bovine serum (FBS, Gibco), 1% penicillin/streptomycin (P/S, Gibco), 1% glutamine (Glu, Gibco), and non-essential amino acids (NEAA, Gibco). C6/36 *Aedes albopictus* and Aag2 *Aedes Aegypti* cells were maintained at 28 °C in a 5% CO_2_ incubator.

### 2.2. Infectious Clone Construction

A modified pSinRep5 plasmid (Invitrogen, Waltham, MA, USA) was used as the backbone for constructing the CHIKV infectious clone. A T7 promoter sequence was introduced upstream of the 5′ end of the CHIKV cDNA, and a poly(A) tail, along with a NotI restriction site, was added to the 3′ end. To avoid introducing mutations during cloning and ensure sequence accuracy, at least two infectious clones of intermediate and final plasmids were selected for full-length sequencing.

### 2.3. Infectious Clone Production

Stbl3 competent cells were transformed with 0.2 μg of plasmid DNA in 20 μL of competent cells by gentle mixing. After incubation on ice for 30 min, the cells were heat-shocked at 42 °C for 90 sec, followed by immediate transfer back to ice for 2–5 min. The transformed cells were then spread onto LB agar plates containing ampicillin (50–100 μg/mL) and incubated at 37 °C for 14–16 h. Single colonies were picked and inoculated into 3–4 mL of LB broth with ampicillin, followed by shaking at 200 rpm at 37 °C for 14–16 h. Plasmid DNA was extracted using the Qiagen Miniprep Kit(Shanghai, China). Prior to transcription, the plasmid was linearized with NotI restriction enzyme (New England Biolabs, Ipswich, MA, USA), and the linearized cDNA template was used for in vitro transcription with the mMESSAGE mMACHINE^®^ T7 High-Yield Capped RNA Transcription Kit (Invitrogen, Waltham, MA, USA).

BHK-21 cells were seeded in 24-well plates at 1 × 10^5^ cells/well and cultured for 12–18 h until 80–90% confluency. For transfection, 2 μg of full-length CHIKV RNA was mixed with 500 μL of Opti-MEM (Thermo Fisher Scientific, Waltham, MA, USA) and 4 μL of LipAofectamine™ 2000 (Invitrogen, Waltham, MA, USA ), incubated at room temperature for 15 min, and then added to the BHK-21 cells. After 6 h, the medium was replaced with fresh DMEM containing 2% FBS. Viral particles were harvested from the supernatant at 36–48 h post-transfection when the cytopathic effect (CPE) became evident. The supernatant was clarified by centrifugation. Furthermore, the infectious clone was passaged twice in C6/36 cells and stored in a −80 freezer for later use.

### 2.4. Viral Infection

Cells were seeded in 24-well plates at a density of 1 × 10^5^ cells per well for most cell types, while C6/36 and Aag2 cells were plated at a higher density of 5 × 10^5^ cells per well to account for differences in growth characteristics. The plates were incubated overnight under appropriate conditions (37 °C with 5% CO_2_ for mammalian cells; 28 °C with 5% CO_2_ for mosquito-derived C6/36 and Aag2 cells) to facilitate cell attachment and formation of a confluent monolayer. After the overnight incubation, the culture medium was aspirated, and cells were infected with the CHIKV infectious clone at a multiplicity of infection (MOI) of 1. The viral inoculum was prepared in infection medium consisting of DMEM supplemented with 2% FBS for most cell lines, whereas MEM with 2% FBS was used for C6/36 and Aag2 cells. Following infection, the plates were incubated for 2 h at 37 °C for mammalian cells or 28 °C for C6/36 cells to allow viral adsorption. Throughout this adsorption period, the plates were gently rocked every 15–20 min to ensure even distribution of the inoculum. After the adsorption period, the viral inoculum was carefully removed, and the cells were washed once with phosphate-buffered saline (PBS) to eliminate any unbound virus. Subsequently, fresh maintenance medium containing 2% FBS was added to each well. At designated time points post-infection, culture supernatants were collected, clarified by centrifugation at 3000× *g* for 5 min to remove cellular debris, and stored at −80 °C until further analysis. Viral titers in the supernatants were determined by plaque assay on Vero cells.

### 2.5. Plaque Assay

Vero cells were seeded in 24-well plates at 1 × 10^5^ cells/well. Overnight, serial 10-fold dilutions of viral samples were prepared in DMEM with 2% FBS, and the cells were infected at 37 °C for 2 h. After removing the inoculum, they were overlaid with DMEM containing 1.3% carboxymethyl cellulose (FUJIFILM Wako, Shanghai, China) and 2% FBS. Two days later, the cells were fixed with formaldehyde and stained with 1% crystal violet to visualize plaques. Viral titers were calculated as plaque-forming units per milliliter (PFU/mL).

### 2.6. Immunofluorescence Assay (IFA)

Cells were seeded in 24- or 96-well plates and infected when 80–100% confluency was achieved. At indicated hours post-infection, the cells were fixed with methanol (100 μL/well for 96-well plates; 350 μL/well for 24-well plates) at −20 °C for 30 min. After fixation, the cells were blocked with 3% BSA at 4 °C overnight, followed by incubation with primary antibody (diluted in 3% BSA) for 90 min at room temperature. After washing with PBS, the cells were incubated with secondary antibody (Invitrogen, diluted in 3% BSA) for 60 min in the dark. Nuclei were counterstained with DAPI (1:5000 in PBS) for 15 min. Fluorescence images were captured using a Cytation 5 (Biotek Instruments, Santa Clara, CA, USA).

### 2.7. Viral Replication Kinetics

Monolayers of BHK-21, U2-OS, C6/36, or other indicated cell lines were infected with virus at a multiplicity of infection (MOI) of 1. Following a 2 h adsorption period at 37 °C (or 28 °C for C6/36 and Aag2 cells), during which the plates were gently rocked every 15–20 min to ensure even viral distribution, the inoculum was removed. The cells were then gently washed once with PBS to eliminate residual unbound viruses. After washing, fresh maintenance medium containing 2% FBS was added to each well. Supernatants were collected at specified time points post-infection (0, 12, 24, 36, 48, and 72 h) and stored at −80 °C until further analysis. Viral titers in the supernatants were subsequently determined by plaque assay performed on Vero cells.

### 2.8. RNA Purification, Reverse Transcription, and RT-qPCR

Total RNA was extracted from infected cells with TRIzol regent (Invitrogen, Waltham, MA, USA) according to the manufacturer’s instructions. The RNA pellets were suspended in 25 μL RNAase-free water and then reverse transcribed with a PrimeScript^TM^ RT Master Mix kit (Takara, Shiga, Japan). Quantitative reverse transcription PCR (RT-qPCR) of virus RNA was performed using SYBR Premix Ex TaqTMII (Tli RNaseH Plus) (TaKaRa, Shiga, Japan). The expression of target genes was normalized to GAPDH mRNA levels in the same samples. The primer sequences for CHIKV are as follows: forward, 5′-ACGCARTTGAGCGAAGCAC-3′; reverse, 5′-CTGAAGACATTGGYCCCAC-3′. The primer sequences for GAPDH are as follows: forward, 5′-TGGGCTACACTGAGCACCAG-3′; reverse, 5′-AAGTGGTCGTTGAGGGCAAT-3′.

### 2.9. Antiviral Compound Evaluation

Vero cells were seeded in 96-well plates at a density of 1 × 10^4^ cells per well and allowed to adhere overnight under standard culture conditions (37 °C, 5% CO_2_). The cells were then infected with virus with test compounds at a final concentration of 40 μM, using a MOI of 0.1. After 2 h of adsorption, the viral inoculum was removed, and the cells were gently washed once with phosphate-buffered saline (PBS) to eliminate unbound virus and compound residues. The wash step was followed by the addition of maintenance medium (DMEM supplemented with 2% fetal bovine serum) containing 40 μM of the respective test compounds. At 24 h post-infection, cell culture supernatants were collected and stored at –80 °C for subsequent viral titration by plaque assay. Meanwhile, the infected cells were fixed with 4% paraformaldehyde for IFA to assess viral antigen expression or other cell-based indicators of infection. The use of a lower MOI (0.1) in the antiviral assays aimed to increase the sensitivity of the assay for detecting compound efficacy, as it allows for more rounds of viral replication.

### 2.10. Data Analysis

Viral titers were determined from at least three independent experiments and presented as mean ± standard deviation (SD). Statistical analysis was performed using GraphPad Prism 10.0, with Student’s test used to compare two variables and the differences in viral replication kinetics between C6/36 and Aag2 cells detected using a two-way ANOVA analysis. *p*-value < 0.05 was considered statistically significant.

## 3. Results

### 3.1. CHIKV Infectious Clone Generation

**Figure 1:**
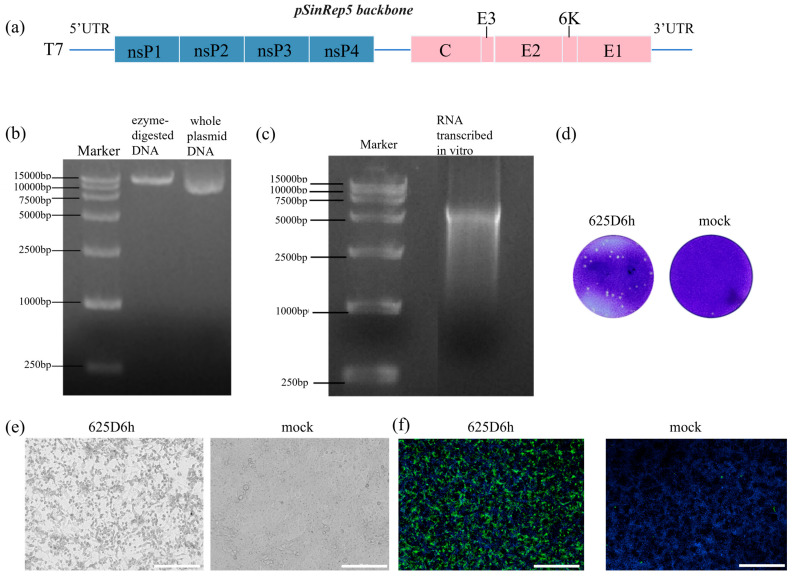
Construction of the infectious clone of CHIKV strain 625D6h. (**a**) Schematic diagram of pSinRep5-625D6h. The full-length genome of 625D6h was synthesized via RT-PCR and assembled into five cDNA fragments, which were then inserted into the pSinRep5 vector containing a T7 promoter to construct the plasmid. The plasmid DNA was linearized by restriction enzyme digestion, followed by in vitro transcription to produce infectious viral particles in transfected cells. The blue and pink regions represent the nonstructural and structural proteins of 625D6h, respectively. (**b**) Electrophoresis results of the constructed CHIKV plasmid and its linearized product. Marker: 250–15,000 bp. (**c**) Electrophoresis results of in vitro transcribed CHIKV RNA. Marker: 250–15,000 bp. (**d**) Plaque formation assay of the infectious clone virus. The supernatant from BHK-21 cells transfected with the 625D6h infectious clone plasmid DNA (**left**) was diluted 10-fold with 6 gradients, and 50 µL of the diluted solution was added to confluent Vero cell cultures in a 24-well plate to observe plaque formation. Uninfected Vero cell cultures are shown on the right. (**e**) Identification of the 625D6h infectious clone. Vero cells were infected with the supernatant obtained from RNA transfection (**left**) or with supernatant containing only transfection reagent (**right**). Cell morphology was observed under a microscope 48 h post-infection. (**f**) Identification of the 625D6h infectious clone. Vero cells were infected as in (**e**) and subjected to immunofluorescence assay using an anti-CHIKV E1 antibody (green signal) and DAPI for nuclear staining (blue signal). Scale bar represents 200 µm.

The synthesized full-genome cDNA fragment of 625D6h was inserted into the pSinRep5 vector, with the T7 promoter located upstream of the viral cDNA (Figure 1a). We amplified the construct pSinRep5-625D6h and selected two monoclonal strains for whole-genome sequencing. Sequence analysis revealed that the region sequences were completely consistent with the 625D6h sequence in the NCBI database (accession number: OK316995.1) [24]. The constructed plasmid (Figure 1b) and the RNA obtained via in vitro transcription (Figure 1c) were verified by gel electrophoresis, with sizes matching the expected results.

The pSinRep5-625D6h construct (2 μg) was transfected into BHK-21 cells in a 24-well plate, and the culture supernatant was harvested 48 h post-transfection. To confirm the successful generation of the 625D6h infectious clone, it was inoculated onto Vero cells; the plaque morphology observed after 2 days is shown in Figure 1d. Additionally, the supernatant obtained from transfection was used to infect Vero cells and obvious CPEs were observed at 48 h post-infection, while mock cells remained normal in morphology (Figure 1e).

An IFA was also performed. The infected cells were stained with virus-specific primary antibodies, followed by corresponding secondary antibodies (green) and DAPI for nuclear staining (blue). As shown in Figure 1f, green fluorescence signals were detected in cells infected with the 625D6h RNA transfection-derived supernatant, whereas no signals were observed in the mock group. The plaque morphology formed by the infectious clone in Vero cells 2 d post-inoculation is shown in Figure 1f. These results demonstrate that the plasmid containing the full-length genome of the 625D6h strain can generate CHIKV infectious clones through in vitro transcription followed by transfection into BHK-21 cells.

### 3.2. Infectious Clone Replication in Mammalian Cell Lines

**Figure 2:**
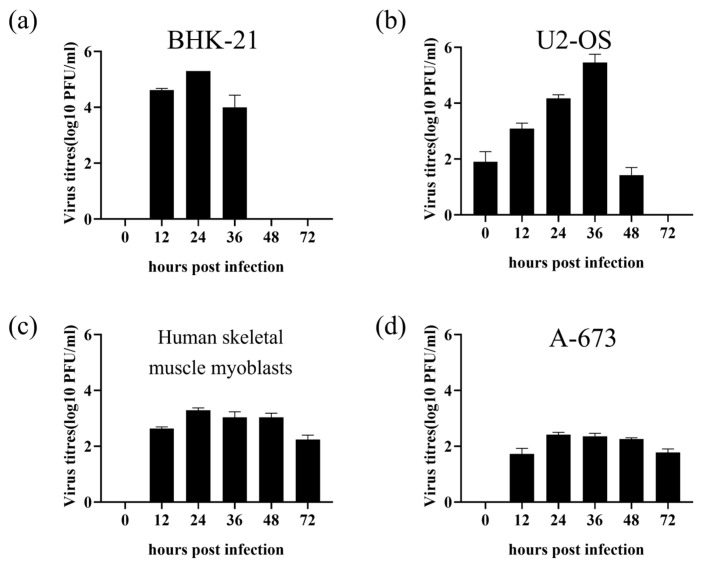
Replication kinetics of the 625D6h infectious clone in mammalian cell lines. BHK-21 (**a**), U2-OS (**b**), Human skeletal muscle myoblasts (**c**) and A-673 (**d**) cells were seeded in 24-well plates at 1 × 10^5^ cells/well and incubated overnight respectively. The cells were then infected with the 625D6h infectious clone (MOI = 1) in DMEM with 2% FBS. After 2 h of incubation at 37 °C, the cells were washed once with PBS and maintained in fresh medium with 2% FBS. Supernatants were collected at 0, 12, 24, 36, 48 and 72 h for viral titer determination by plaque assay. Each experiment was repeated three times biologically and the results shown are the averages (n = 3), with error bars indicating standard deviation.

We evaluated the replication efficiency of the CHIKV 625D6h infectious clone in BHK-21, U2-OS, human skeletal muscle myoblasts and A-673 cell lines. The tested cells were seeded at 1 × 10^5^ cells/well in 24-well plates and incubated overnight before infection with the 625D6h infectious clone at an MOI of 1. Supernatants were collected at 0, 12, 24, 36, 48 and 72 h post infection [27,28]. Viral titers in the culture supernatant were determined by plaque assay. The results showed that, in BHK-21 cells, the viral titer peaked at 24 h post-infection, reaching approximately 2.6 × 10^5^ PFU/mL (Figure 2a). U2-OS cells supported viral replication up to 48 post-infection, with the titer peaking at 36 hpi (2.8 × 10^5^ PFU/mL) (Figure 2b), while the virus titers were about 10^2^–10^4^ PFU/mL for human skeletal muscle myoblasts and A-673 cells (Figure 2c,d).

**Figure 3:**
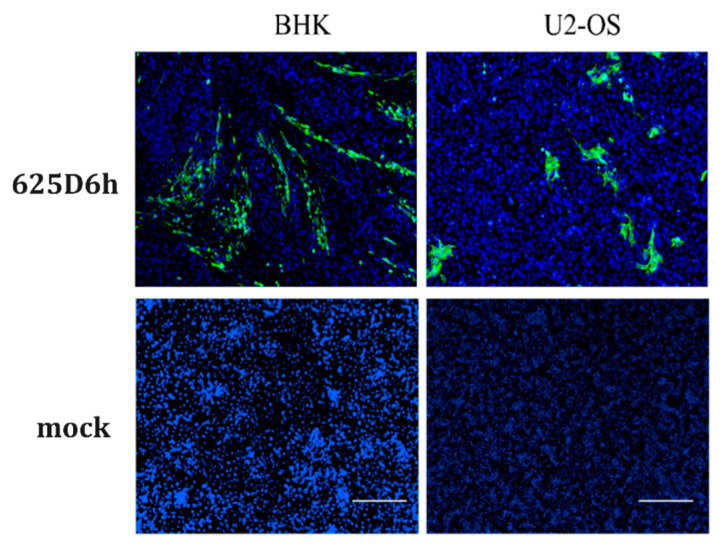
Immunofluorescence detection of CHIKV replication. BHK-21 (**left panel**) and U2-OS cells (**right panel**) were exposed to the 625D6h infectious clone (**upper row**), with unexposed controls (**lower row**). At 24 h post-infection, cells were stained with anti-CHIKV E1 antibody (green signal) and DAPI (blue signal). (Scale bar, 200 μm).

To visualize viral infection, BHK-21 and U2-OS cells were infected with the virus at an MOI of 1 and immunofluorescence staining was performed at 24 h post-infection. Green fluorescent signals were observed in the infected cells (Figure 3), confirming the successful construction of the infectious clone. Under optical microscopy, extensive cell death was observed in BHK-21 cells infected with 625D6h, consistent with previous reports that CHIKV infection induces significant cytopathic effects in mammalian cells [29,30].

### 3.3. Evaluation of Replication Efficiency in C6/36 and Aag2 Cells

**Figure 4:**
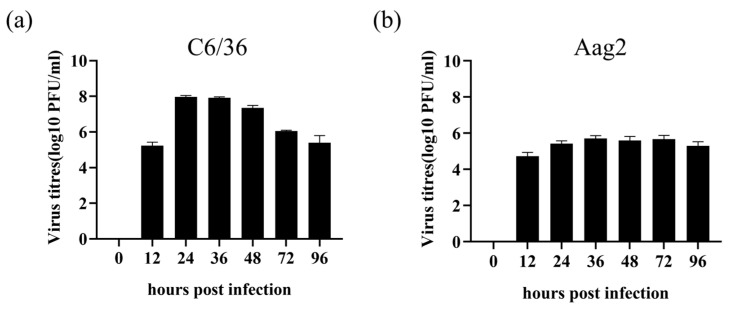
Replication kinetics of 625D6h infectious clone in C6/36 and Aag2 cells. C6/36 (**a**) or Aag2 (**b**) cells were seeded in 24-well plates at a density of 5 × 10^5^ cells/well. Overnight, the cells were then infected with the 625D6h infectious clone (MOI = 1) in DMEM with 2% FBS. After 2 h of incubation at 28 °C, the cells were washed once with PBS and maintained in fresh medium with 2% FBS. Supernatants were collected at 0, 12, 24, 36, 48, 72 and 96 h for viral titer determination by plaque assay. Each experiment was repeated three times biologically and the results shown are the averages (n = 3), with error bars indicating standard deviation. The differences in viral replication kinetics between C6/36 and Aag2 cells were detected using a two-way ANOVA analysis.

To assess the replication of the CHIKV infectious clone in the C6/36 *Aedes albopictus* and Aag2 *Aedes Aegypti* cells, these were seeded in 24-well plates at a density of 5 × 10^5^ cells/well. After overnight incubation, they were infected with 625D6h at an MOI of 1. Supernatants were collected at multiple time points and viral titers were determined by plaque assay. Two-way ANOVA analysis revealed that the virus titers in the supernatant harvested from C6/36 cells infected with the 625D6h strain at the 0–96 h stage were significantly higher than those in Aag2 cells (*p* value < 0.001). The 625D6h strain reached its highest titer (10^8^ PFU/mL) at 36 h post-infection in C6/36 cells (Figure 4a), whereas the highest titer was less than 10^6^ PFU/mL for Aag2 cells (Figure 4b). The results demonstrated that the 625D6h strain replicates more efficiently in C6/36 cells. Unlike in mammalian cells, optical microscopy observation showed that CHIKV infection in C6/36 and Aag2 cells did not induce significant CPEs [30].

### 3.4. Antiviral Compound Efficacy Evaluation

**Figure 5:**
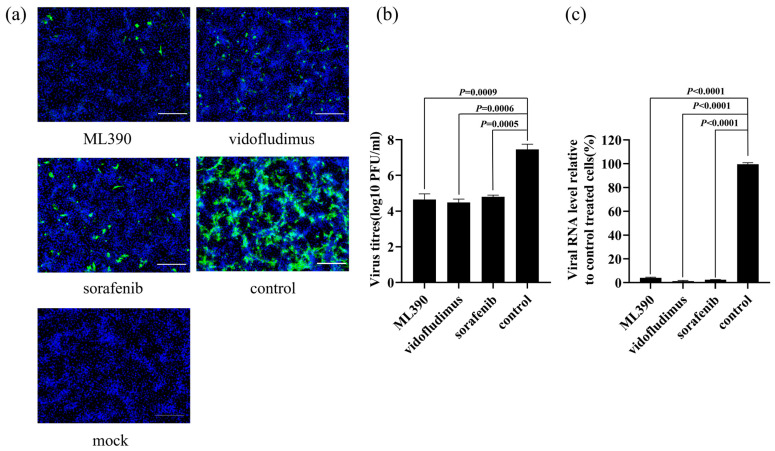
Inhibition of 625D6h replication by ML390 and vidofludimus. 1 × 10^4^ Vero cells were seeded in 96-well culture plates. Overnight, the cells were infected with 625D6h infectious clone at different MOI of 0.1 and treated with dimethyl sulfoxide (DMSO, control group) or 40 μM of compounds. At 24 h post-infection, infected cells were analyzed by IFA (Scale bar, 200 μm) (**a**), and cell supernatants were collected and virus titer was quantified using plaque assay (**b**) and cell supernatants were collected and virus mRNA expression was quantified using RT-qPCR (**c**). The known anti-CHIKV compound sorafenib served as a positive control. The control group were infected with 625D6h infectious clone and treated with dimethyl sulfoxide. The mock group were not infected with 625D6h infectious clone. Each experiment was repeated three times biologically. Statistical significance was determined using an unpaired *t*-test (Control vs. treated group). The results shown are the averages (n = 3), with error bars indicating standard deviation.

Our previous studies identified two dihydroorotate dehydrogenase (DHODH) inhibitors, ML390 and vidofludimus, which exhibited significant antiviral effects against West Nile virus [31]. Given the crucial role of DHODH in viral nucleic acid replication, we hypothesized that these compounds might also inhibit CHIKV replication. To test this hypothesis, we conducted in vitro antiviral assays using sorafenib—a known anti-CHIKV compound—as a positive control [32]. The results demonstrated that, compared to the control group, both ML390 and vidofludimus treatment groups showed a significant reduction in viral fluorescence signal intensity and the viral titres of supernatants (*p* value < 0.001). These findings indicate that the DHODH inhibitors—vidofludimus and ML390—can effectively suppress CHIKV replication, further supporting DHODH’s potential as an antiviral target (Figure 5a,b). This discovery provides important experimental evidence for the development of novel antiviral drugs against CHIKV.

## 4. Discussion

In 2019, the CHIKV strain 625D6h was isolated from a dengue-like febrile patient at the People’s Hospital of Xishuangbanna Dai Autonomous Prefecture in Yunnan Province, China. To explore the biological significance of these mutations, this study constructed an infectious clone of the 625D6h strain. In vitro experiments showed that this infectious clone exhibited limited replication levels in mammalian cell lines (such as Vero or BHK-21) but was accompanied by significant cytopathic effects (CPEs).

**Figure 6:**
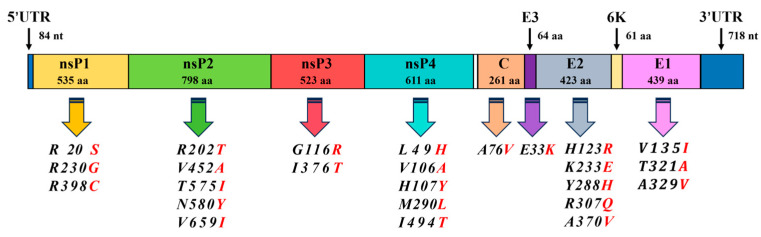
Amino acid mutations of 625D6h compared to SZ1239. Compared to the SZ1239 strain, the 625D6h strain has accumulated 25 amino acid mutations (Red mark).

Whole-genome sequencing and evolutionary analysis revealed that this strain belongs to the Asian genotype and is most closely related to the SZ1239 strain (GenBank: MG664851.1), which was isolated in July 2012 from a Chikungunya case (originating from Southeast Asia) imported from Shenzhen. The nucleotide homology between the two strains reached 99.2%. Compared to the SZ1239 strain, the 625D6h strain has accumulated 25 amino acid mutations distributed across viral genomes (Figure 6). Further analysis of the potential mutation site functions revealed that the five mutations in the E2 protein are located near known mosquito receptor-binding domains, while the three in the E1 protein are associated with membrane fusion functions. These mutations may have synergistic effects with previously reported host-adaptive mutations [33,34,35].

While direct causal inference requires further investigation, our in vitro finding of enhanced replication in mosquito cells presents a plausible virological trait that could be contextualized within the epidemiology of the 2019 Yunnan outbreak. Entomological surveys from the affected region identified *Aedes albopictus* as the primary vector, a species highly susceptible to CHIKV. Furthermore, the simultaneous circulation of Dengue virus in Yunnan creates a potential for vector competition; a virus with a replicative advantage might achieve more efficient vector infection. Our data do not prove this strain drove the outbreak. However, they provide a testable molecular hypothesis for its contribution, that these mutations may have enhanced fitness in the local mosquito vector population, thereby facilitating the observed intense and rapid transmission dynamics. The association between this replicative characteristic and the 2019 Yunnan outbreak remains speculative and is presented here merely as a hypothetical framework for further investigation. Future studies involving in vivo vector competence experiments with local *Aedes albopictus* populations and genomic epidemiology tracking these mutations across outbreak strains are needed to substantiate this link.

The 625D6h infectious clone, exhibiting enhanced replication in mosquito cells but attenuated replication in mammalian cells, holds significant promise for both vaccine development and fundamental research. It can be used to investigate the molecular determinants of host tropism and viral replication, elucidating why certain mutations enhance growth in arthropod cells while restricting it in mammalian systems. This knowledge is crucial for understanding CHIKV pathogenesis and host adaptation. We are constructing an infectious clone of the strain SZ1239 to compare the biological properties of the two strains, aiming to clarify the contribution of key mutations to host switching.

In summary, this study provides the first detailed insight into the molecular characteristics and potential host-adaptive mechanisms of the locally circulating CHIKV strain 625D6h in southwestern China. A significant limitation of this study is the unavailability of the parental clinical isolate for direct comparison. While our genomic and phenotypic characterization provides a detailed snapshot of the laboratory clone, the conclusions regarding adaptation are drawn specifically for the clone used in this study within the context of its laboratory environment. Furthermore, the work is limited by its reliance on in vitro approaches, lacking complementary field epidemiological data and in vivo validation. Future studies will focus on integrating ecological and animal model experiments to further substantiate these findings.

## Data Availability

The data that support the findings of this study are available from the corresponding author upon reasonable request.
